# Roles of Integrin α6β4 Glycosylation in Cancer

**DOI:** 10.3390/cancers9070079

**Published:** 2017-07-05

**Authors:** Yoshinobu Kariya, Yukiko Kariya, Jianguo Gu

**Affiliations:** 1 Department of Biochemistry, Fukushima Medical University School of Medicine, 1 Hikarigaoka, Fukushima City, Fukushima 960-1295, Japan; ykk-kari@fmu.ac.jp; 2 Division of Regulatory Glycobiology, Institute of Molecular Biomembrane and Glycobiology, Tohoku Medical and Pharmaceutical University, 4-4-1 Komatsushima, Aoba-ku, Sendai, Miyagi 981-8558, Japan

**Keywords:** integrin, glycosylation, cancer, *N*-acetylglucosaminyltransferase-V (GnT-V), epithelial to mesenchymal transition (EMT), galectin-3

## Abstract

Malignant transformation is accompanied with aberrant glycosylation of proteins. Such changes in glycan structure also occur in the integrins, which are a large family of cell surface receptors for the extracellular matrix and play key roles in tumor progression. There is now increasing evidence that glycosylation of integrins affects cellular signaling and interaction with the extracellular matrix, receptor tyrosine kinases, and galectins, thereby regulating cell adhesion, motility, growth, and survival. Integrin α6β4 is a receptor for laminin-332 and the increased expression level is correlated with malignant progression and poor survival in various types of cancers. Recent studies have revealed that integrin α6β4 plays central roles in tumorigenesis and the metastatic process. In this review, we summarize our current understanding of the molecular mechanisms of tumor progression driven by integrin α6β4 and also discuss the modification of glycans on integrin β4 subunit to address the important roles of glycan in integrin-mediated tumor progression.

## 1. Introduction

Integrins are a large family of heterodimeric transmembrane receptors comprising α and β subunits. In mammals, 18α and 8β subunits have been characterized, and the combination of them forms 24 distinct integrins. Integrins bind to extracellular matrix proteins including collagen, fibronectin, laminin, osteopontin, and tenascin in the extracellular domain, which leads to the assembly of signaling complexes including focal adhesion kinase, paxillin, and Src in the cytoplasmic domain and the rearrangement of actin cytoskeleton. The transducing signals from integrin receptors to cytoskeletal and adhesive machinery regulate cell adhesion, migration, proliferation, differentiation, and tumor progression.

Of note, integrins are known to be major glycan-carrying proteins. In fact, the functions of integrins are also dependent on their complex *N*-glycosylation modifications [1]. Among the different types of integrins, α5β1, a major fibronectin receptor, is believed to be a relatively well-characterized example, and *N*-glycosylation is important for its mediated many biological functions such as cell adhesion and migration [2,3]. Alterations in the oligosaccharide portion of integrin α5β1 from the enhanced expression of some glycosyltransferase genes—such as *N*-acetylglucosaminyltransferase-V (GnT-V), *N*-acetylglucosaminyltransferase-III (GnT-III), or α2,6-galactoside sialyltransferase 1—can be used to regulate the cell spreading and migration onto fibronectin [1,4,5]. Furthermore, we recently found that *N*-glycosylation on the calf domain of α5, putative sites 10–14, was essential for the α5-mediated inhibitory effect on epidermal growth factor receptor (EGFR) signaling and cell proliferation [6], while *N*-glycosylation on sites 1–2 on the β-propeller domain of α5 played a key role in driving integrin α5β1 dynamics and cell migration [7]. Taken together, these findings support the idea that individual integrin α5 *N*-glycosylation differentially functions as a molecular switch to regulate the biological functions of α5β1. Similarly, recent studies have revealed that glycosylation of the integrin β4 subunit is important for integrin α6β4 functions, the expression of which is associated with cancer progression. In this review, we summarize our current understanding of integrin α6β4 in cancer and also discuss function of glycans on integrin β4 subunit.

## 2. Structure and Functions of Integrin α6β4

**Figure 1:**
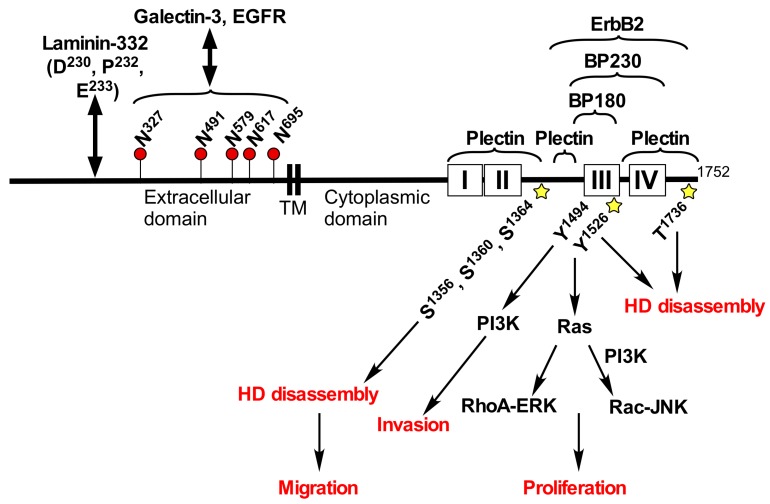
Structure and functions of integrin β4. Integrin β4 contains laminin-332 binding sites [14] and five *N*-glycosylation sites (Asn^327^, Asn^491^, Asn^579^, Asn^617^, Asn^695^) in its extracellular domain [55], and the binding sites for Plectin [70], BP180, BP230 [71,72], and ErbB2 [45] in its cytoplasmic domain. Phosphorylation of Ser^1356^, Ser^1360^, Ser^1364^, Tyr^1526^, and Thr^1736^ induces hemidesmosome disassembly [19,37,42,43]. Phosphorylation of Tyr^1526^ promotes recruitment of Shc, which in turn activates Ras, Raf-ERK and Rac-JNK signaling [31,37]. Tyr^1494^ is associated with PI3K activation [33]. *N*-Glycosylation sites are shown by flags. Numbers and boxes indicate the number of amino acid residue and the four fibronectin type III repeats, respectively. Star shape indicates phosphorylation site. TM, transmembrane region. HD, hemidesmosome. EGFR, epidermal growth factor receptor.

Integrin α6β4 is an essential component of the hemidesmosome that provides stable adhesion of basal epithelial cells to the underlying basement membrane [8,9,10]. The integrin β4 can form heterodimer only with the α6 integrin. Patients with genetic mutations in either integrin α6 or β4 subunit suffer from the junctional epidermolysis bullosa with pyloric atresia (JEB-PA), which is an autosomal-recessive disorder clinically characterized by mucocutaneous fragility and gastrointestinal atresia [11,12,13]. The extracellular domain of integrin β4 associates with extracellular matrix, laminin-332, which is a major component of the hemidesmosome [14,15] (Figure 1). The cytoplasmic domain of integrin β4 is much longer (>1000 amino acid) than that of other integrin β subunits (<50 amino acid) [16], and the large cytoplasmic domain of integrin β4 interacts with other hemidesmosome component, plectin, collagen XVII (BP180/BPAG2), and BP230 (BPAG1) [9,10] (Figure 1). The adhesion complex consisting of those hemidesmosome proteins plays an important role in maintaining the hemidesmosome structure. Mice carrying a target deletion of the integrin β4 cytoplasmic domain display extensive epidermal detachment at birth and die shortly thereafter from a syndrome resembling the human JEB-PA [17]. The integrin β4 cytoplasmic domain contains several serine, threonine, and tyrosine phosphorylation sites (Figure 1), and the phosphorylation of integrin β4 cytoplasmic domain is caused by activation of receptor tyrosine kinases (RTKs) [18], and directly by protein kinase [19].

## 3. Integrin α6β4 in Cancer

Integrin α6β4 was first discovered as a tumor-specific antigen [20,21]. Subsequent studies demonstrated that increased expression level of integrin α6β4 was correlated with malignant progression and poor survival in squamous cell carcinoma (SCC) of the skin [21,22], lung [23], head and neck [24], and cervix [25]. Further studies have reported that high expression levels of integrin α6β4 were found in several types of cancer—including breast, bladder, colon, ovarian, pancreatic, prostate, and thyroid—and linked to poor prognosis [26]. In a mouse model of active H-Ras and IκBα-driven human cutaneous SCC, integrin β4-negative keratinocytes (derived from JEB-PA patients with null ITGB4 gene mutations) failed tumor formation but reintroduction of integrin β4 gene into the cells restored it [27], suggesting that integrin β4 plays an essential role in human SCC development.

Association of integrin α6β4 with laminin substrates significantly promotes cancer cell adhesion, migration, invasion, proliferation, and tumorigenesis through the activation of Rac1, PKC, PI3K, and ERK signaling pathways [10,14,26,28,29,30,31,32] (Figure 1). The PI3K activation response to integrin α6β4 ligation is involved in invasive potential of carcinoma cells, and Tyr^1494^ in the cytoplasmic domain of the integrin β4 is required for the activation [33]. Ligand binding to the extracellular domain of integrin α6β4 induced phosphorylation at serine and tyrosine residues in integrin β4 cytoplasmic domain, which were associated with a metastatic phenotype of cancer cells [34]. Phosphorylation of Tyr^1494^ and Tyr^1526^ in integrin β4 leads to recruitment of tyrosine phosphatase Shp2 and Shc to the β4 cytoplasmic domain, respectively, followed by activation of Ras-MAP kinase pathways, and promotes cell cycle progression [31,35,36,37] (Figure 1). However, crystallographic studies have been shown that the structural environment of Tyr^1494^ and Tyr^1526^ are not compatible with binding to the SH2 and PTB binding domains of Shp2 and Shc, respectively [38]. Furthermore, both Tyr residues are not well solvent-exposed. It is therefore questionable whether these residues are involved in the recruitment of SHP2 and Shc, and the subsequent coupling of the integrin α6β4 to the MAPK signaling pathways [39].

During cancer progression, integrin α6β4 is released form hemidesmosomes and the number of hemidesmosomes is decreased, which facilitates the cancer cell migration and invasion [26,39]. Serine phosphorylation of integrin β4 cytoplasmic domain by PKC induces relocation of integrin α6β4 from hemidesmosomes to cell protrusions in cancer cells [40]. Compared with carcinoma in situ or normal tissue, increased phosphorylation at Ser^1356^ in the integrin β4 cytoplasmic domain was found in around 60% of invasive cutaneous SCC. Triple mutation at Ser^1356^, Ser^1360^, and Ser^1364^ to non-phosphorylatable alanines in the integrin β4 cytoplasmic domain stabilized hemidesmosome-like structures and reduced cell migration in SCC cells [41]. Thus, the phosphorylation at specific sites in the integrin β4 cytoplasmic domain leads to the disruption of stable adhesion structure, hemidesmosomes [19,37,42,43], thereby facilitating the migration of cancer cells (Figure 1). Integrin β4 is also phosphorylated by the associations with several RTKs—including EGFR, ErbB2, and Met [18,32]—which are often mutated or amplified in tumors. RTKs activate Src-family kinases, and thereby phosphorylates integrin β4 cytoplasmic domain. Tyrosine phosphorylation of integrin β4 through Src family kinase, Fyn, which is activated by EGFR, causes disruption of hemidesmosomes, thereby promoting squamous carcinoma invasion [44]. Conversely, integrin α6β4 regulates the expression of ErbB2 and the subsequent Src-family kinase-dependent phosphorylation of RTKs and activation of Ras, STAT-3, and c-Jun [45]. These findings suggest that cooperative signaling between integrin β4 and RTKs promotes cancer progression.

Metastasis of cancer cells is a major cause of death in patients with cancer. A first step in metastasis of cancer cells is to move from the primary site and invade into the stroma. In the process of metastasis, some cancer cells undergo epithelial to mesenchymal transition (EMT), which is characterized by loss of epithelial phenotype with cell-cell adhesion and cell polarity, and gain of fibroblast-like morphology [46]. EMT induces cell motility, and stem cell-like properties, thereby enhancing cancer invasion, metastasis, and chemoresistance [47]. A cDNA microarray analysis using clinical samples of pancreatic ductal adenocarcinoma revealed that high levels of integrin β4 expression were significantly correlated with the hallmarks of EMT, with high tumor grade, and with the presence of lymph node metastasis [48]. Overexpression of integrin β4 promoted cell motility of pancreatic ductal adenocarcinoma cell lines in combination with down-regulation of E-cadherin and up-regulation of vimentin expression [48]. Integrin α6β4 also promotes EMT in hepatocellular carcinoma by upregulating the expression of transcription factor Slug that inhibits the transcription of E-cadherin gene [49]. A recent report has demonstrated that cells with an intermediate level of integrin β4 expression exhibited a hybrid epithelial/mesenchymal phenotype and contained cancer stem cell-enriched populations in triple-negative breast cancer cells. Therefore, integrin β4 can be a mechanistically driven prognostic biomarker for identifying the more aggressive subtypes of mesenchymal carcinoma cells in triple-negative breast cancer cells [50]. A subpopulation of the PC-3 prostate cancer cell line, TEM4-18, displayed the hallmarks of EMT, including frank loss of E-cadherin expression and upregulation of E-cadherin repressor ZEB1 compared to parent cells [51]. Surprisingly, the ZEB1-mediated EMT in TEM4-18 cells repressed integrin β4 and laminin-332 expression by the binding of ZEB1 to the promoter elements of integrin β4 and laminin γ2 (one of the subunit of laminin-332) genes. The ZEB1 expression exhibited enhanced trans-endothelial migration but decreased transwell migration and invasion of cancer cells [51]. These results suggest that integrin α6β4 is associated with EMT, but the regulatory mechanism of EMT by integrin α6β4 might depend on cancer types.

Exosomes are cell-derived small membrane vesicles (30–100 nm) containing proteins, lipids, RNA, and DNA that can be horizontally transferred to recipient cells [52]. Recent evidence suggests that exosomes play a critical role in the development of cancers, such as activation of fibroblasts, promoting angiogenesis, enhancing invasiveness and chemoresistance [52]. Hoshino et al. have reported that exosomes containing integrin α6β4 and αvβ5 derived from tumor cells were associated with lung and liver metastasis, respectively [53]. Furthermore, exosomal integrin α6β4 uptake activated Src and upregulated pro-migratory and pro-inflammatory S100 molecules in resident cells. These results suggest that exosomal integrin α6β4 determines metastatic organotropism and could be a biomarker for lung-specific metastasis.

## 4. Roles of Glycans in Integrin β4 Function

*N*-glycosylation is a common protein post-transcriptional modification occurring on asparagine in the asparagine-X-serine/threonine motif, where X can be any amino acid except proline. Integrins α6 and β4 have nine (Asn^78^, Asn^223^, Asn^284^, Asn^370^, Asn^731^, Asn^748^, Asn^891^, Asn^927^, Asn^958^) [54] and five (Asn^327^, Asn^491^, Asn^579^, Asn^617^, and Asn^695^) *N*-glycosylation potential sites in each extracellular domain, respectively [55] (Figure 1). Although the *N*-glycans on integrin β1 is required for the heterodimer formation with integrin α5 [56], the presence of *N*-glycans on integrin β4 is not essential for integrin α6β4 heterodimer formation [55]. In contrast, a defect of *N*-glycosylation in integrin β4 decreases its function such as cell spreading, adhesion, and migration on its substrate, laminin-332, as well as localization to lipid rafts [55].

**Figure 2:**
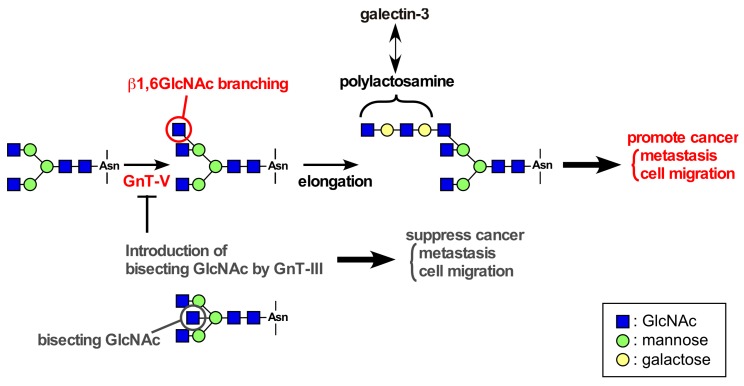
Glycosylation reactions catalyzed by GnT-V. GnT-V catalyzes the formation of β1,6-GlcNAc-branched structures. β1,6-GlcNAc-branching can be elongated with *N*-acetyllactosamine repeats (polylactosamine), which acts as a high-affinity ligand for galectin-3. Enhanced expression of GnT-V results in increased migration and metastasis of cancer cells. GnT-III adds GlcNAc to the core mannose to form bisecting *N*-acetylglucosamine (GlcNAc) in *N*-glycans, which inhibit the β1,6-GlcNAc branching formation catalyzed by GnT-V and the resultant increase in cancer migration and metastasis.

Overexpression of β1,6-*N*-acetylglucosamine (GlcNAc)-branched *N*-glycans is often found in tumor tissues, and the increase in β1,6-GlcNAc-branched *N*-glycans is directly associated with malignancy and poor prognosis [57]. The addition of the β1,6-GlcNAc-branched *N*-glycans is catalyzed by GnT-V, a member of the family glycosyltransferase [58] (Figure 2). GnT-V knockout mice showed reduced β1,6-GlcNAc-branched *N*-glycans, resulting in suppression of mammary tumor growth and metastasis induced by the polyomavirus middle T oncogene [59]. In vitro, β1,6-GlcNAc-branched *N*-glycans-modified integrins α3β1 and α5β1, and laminin-332 strongly promoted cancer cell motility [60,61,62]. In contrast, introduction of bisecting GlcNAc by GnT-III expression suppresses β1,6-GlcNAc branching formation catalyzed by GnT-V [58], resulting in suppression of cancer metastasis (Figure 2). These findings indicate that β1,6-GlcNAc-branched *N*-glycans catalyzed by GnT-V play important roles in tumor malignancy and progression.

Galectins are a family of soluble lectins that bind β-galactoside-containing glycans such as *N*-acetyllactosamine (Galβ1,4-GlcNAcβ1,3). The most studied member of the galectin family, galectin-3 is known to be associated with cancer aggressiveness and metastasis [63,64]. The binding of galectin-3 to β-galactoside sugars on glycoproteins crosslinks between the glycoproteins and regulates diverse cellular functions in cancer cells. β1,6-GlcNAc-branched *N*-glycans catalyzed by GnT-V can be elongated with *N*-acetyllactosamine repeats (polylactosamine), which acts as a high-affinity ligand for galectin-3 (Figure 2). Previously, we found the molecular complex consisting of integrin α6β4, EGFR, and galectin-3 in gastric cancer cell line MKN45 cells, which highly express GnT-V [65,66]. The formation of integrin α6β4/EGFR/galectin-3 complex was inhibited by either the presence of a competitive inhibitor of galectin-binding to β-galactoside structure, β-lactose or GnT-III expression [66]. In addition, the breakdown of the tri-molecular complex by an anti-galectin-3 antibody inhibited integrin α6β4 clustering and cell migration [66]. Similar effect was also observed on the laminin-332/integrin α6β4 association. In GnT-III-overexpressing MKN45 cells, the modification of laminin-332 increased bisecting GlcNAc, thereby decreasing β1,6-GlcNAc branched *N*-glycans, as well as integrin α6β4 clustering and cell motility [61]. These findings indicate that galectin-3 cross-links among integrin α6β4, EGFR, and laminin-332, thereby inducing efficient signaling and the following cellular function.

Mucin type *O*-glycosylation (hereafter referred to as *O*-glycosylation) is one of the most abundant forms of post-translational modification of secreted and membrane-bound proteins that contains a range of *N*-acetylgalactosamine (GalNAc)-Serine/Threonine *O*-linked oligosaccharaides (*O*-glycans) [67]. Sialic acids occupy terminal positions of *N*-glycans and *O*-glycans in glycoproteins, and altered sialylation has long been associated with the cancer progression. Desialylation of *O*-glycans on integrin β4 by sialidase NEU1 suppressed colon cancer cell adhesion to laminin-332, tyrosine phosphorylation of integrin β4, and metastasis of human colon cancer cells [68]. In contrast, sialylation of integrin β4 was downregulated during EMT but then reverted and upregulated in the mesenchymal state after EMT [69]. These results indicate that sialylation of integrin β4 is dynamically regulated and contributes to cancer progression. Although there are some data using lectin suggesting that *O*-glycosylation may occur on the integrin β4 [55,68], direct evidence for the *O*-glycan structure and *O*-glycosylation site in the molecule has not been presented. Further studies including mass spectrometry analysis are required for the study about *O*-glycosylation on the integrin β4.

## 5. Conclusions and Perspective

In normal stratified and complex epithelial tissues, integrin α6β4 is an essential component of the hemidesmosomes. However, integrin α6β4 also overexpresses in several types of cancers and the expression level is correlated with malignant progression and poor survival in cancer patients. Integrin α6β4 significantly promotes cancer cell adhesion, migration, invasion, proliferation, and tumorigenesis through the activation of Rac1, PKC, PI3K, and ERK signaling pathways, which are induced by the interaction with other molecules including RTKs and laminin-332. Phosphorylation of the cytoplasmic domain in integrin β4 also contributes to the cancer progression by activation of Ras-MAP kinase pathways and hemidesmosome disassembly. In addition, the expression levels of integrin β4 are closely correlated with the hallmarks of EMT, and also exosomal integrin α6β4 determines metastatic organotropism.

The biosynthesis of glycan is primarily determined by the glycosyltransferases, the expression level of which is controlled at the level of gene transcription, and by enzymatic activity and chaperone. Since the expression profile of glycolsyltransferases in cancer cells is quite different from that of normal cells, the resultant glycan structure is aberrant and specific to cancer. Therefore, alteration of glycan structures is one of the hallmarks of cancer. Recent studies have revealed that integrin α6β4 functions are regulated by glycosylation of integrin β4. The formation of integrin α6β4/EGFR/galectin-3 complex through *N*-glycans induces integrin α6β4 clustering and cell migration. Specifically, sialylation of integrin β4 seems to be associated with cancer progression. Therefore, glycosylation on integrin β4 may be a useful biomarker and a novel therapeutic target for cancer.
